# Trends and causes of maternal mortality in a tertiary care hospital over five years: 2013-2017

**DOI:** 10.12669/pjms.35.4.1091

**Published:** 2019

**Authors:** Sonia Rafiq, Wajeeha Syed, Simi Fayaz Ghaffar

**Affiliations:** 1 Dr. Sonia Rafiq, FCPS. Department of Obstetrics & Gynaecology, Lady Reading Hospital, Peshawar, Pakistan; 2 Dr. Wajeeha Syed, FCPS. Department of Obstetrics & Gynaecology, Lady Reading Hospital, Peshawar, Pakistan; 3 Dr. Simi Fayaz Ghaffar, FRCOG, FCPS. Department of Obstetrics & Gynaecology, Lady Reading Hospital, Peshawar, Pakistan

**Keywords:** Maternal mortality, Maternal mortality rate, Hemorrhage, Hypertension

## Abstract

**Background and Objective:**

Maternal death measurement is essential to a country’s wellbeing and development status. In emerging countries like Pakistan, it remains an intimidating and failed public health challenge. Objectives of our audit were to estimate trends and causes of maternal demise in Lady Reading Hospital, Peshawar, Pakistan.

**Methods:**

Between January 2013 to December 2017, a retrospective study was carried out at Medical Teaching Institute, Lady Reading Hospital, Peshawar. A structured proforma was used to collect data from the medical records. To detect trends in mother demise maternal mortality ratio was calculated for each year and for all five years, Spss version 23 was used for data analysis.

**Results:**

In the five-year periods 134 deaths were recorded. The maternal mortality during the study period was 431/100,000 live births. An unstable trend in mortality with two crowning periods in 2013 and 2017 was observed. Hemorrhage persisted as the foremost cause of maternal death over the five years period, accounting for 47.76% deaths followed by hypertension, accounting for 25.37% deaths. An increased risk of 35.08% was observed among women aged 25-29 years, followed by 26.11% in 20-24 years and 23.88% in >30 years.

**Conclusion:**

There is a decreasing trend of maternal death from 2013 to 2016 but a slight increase was noted in 2017. Hemorrhage was the top cause responsible for the maternal death.

## INTRODUCTION

Maternal mortality is defined by World health organization (WHO) as the demise of a woman during maternity in the first 42 days of cessation of pregnancy, regardless of site & time of the pregnancy, from any reason linked to gravidity or its management, but not from unintentional causes. In the emerging countries like Pakistan maternal health has become one of the main public health worries after the first safe motherhood meeting held in Kenya in 1987.^1^ Still, maternal mortality is at the top of a global challenge with more than 0.3 million deaths take place due to pregnancy and its complication in the year 2015. A target was set by the millennium development goal to reduce MMR by 75% in the year 2015.^2^ During the year 2013 MMR in developed and developing countries was 16/100,000 and 230/100,00 live births respectively in which chief input of the global maternal deaths was from African region alone (62%) followed by Southern Asia (24%).^3^

In 2013 MMR has declined in Pakistan from 400 in 1990 to 230 in 2013, currently, Pakistan is off-track and lags behind the target (140) set for 2015.^4^ Numerous native studies have shown mutable figures for MMR from dissimilar parts of the study.^5^,^6^ Haemorrhage is the leading cause of maternal deaths.^7^ Poverty, Meagre access to healthcare facilities, lack of skilled primary health care providers and local custom and fondness of home deliveries are contributing towards higher mortalities in Pakistan.^8^

Our study was commenced to find the trends in the maternal mortality in our hospital and to determine the major causes of death and to ascertain if there are any significant deviations from other reports.

## METHODS

This retrospective study of maternal deaths was carried out from January 2013 to December 2017 at Medical Teaching Institute, Lady Reading Hospital, Peshawar, Pakistan. Data collection tool was a self-structured proforma, on which required data was collected from the medical records the medical records of maternal deaths were studied. Data on all the cases was haul out from the Labour unit register, patient’s case notes and maternal mortality records. From the delivery register demographic data, total deliveries and live birth for the period were noted.

Only those females were counted in who acceded to death during and after delivery, rest all morbidity cases were excluded from the study. The patients who reach hospital in emergency condition or dead while reaching were excluded. Consent was taken from Medical Superintendent of the hospital and Head of Department of Gynaecology/ Obstetrics ward, Leady Reading Hospital, Peshawar. Privacy was ensured regarding the collected data that it was chastely for research purposes and will not be public with the third party. The Ethical Review Board of Lady Reading Hospital, Pakistan approved the study.

Statistical analysis was achieved by using the Statistical Package for Social Science (SPSS) version 23. Continuous data were showed as the mean ± standard deviation, while the categorical and nominal data were presented as frequencies and percentages.

## RESULTS

**Fig. 1:**
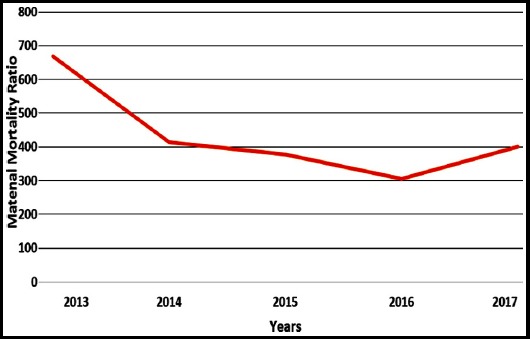
Trends in maternal mortality over five years.

A total of 134 deaths were logged in five years study period. The MMR during the study period was 431/100,000 live births as shown in Table-I. A changing trend in MMR with two peak periods in 2013 and 2017 was detected as shown in Fig.1. Hemorrhage remained the principal cause of maternal mortality over the five years period, accounting for 64(47.76%) deaths followed by hypertension accounting 34(25.37%) deaths. Sepsis leads to 06(4.47%) maternal deaths. Table-II shows all the direct and indirect causes of maternal mortality. An increased risk of 35.08% was observed among women aged 25-29 years, followed by 26.11%) in 20-24 years and 23.88% in >30 years, depicted in Table-III.

**Table I:** Trends in maternal mortality 2013-2017.

	2013	2014	2015	2016	2017	Total
Total birth	7435	6400	7892	7684	7847	37,258
Live birth	6124	5550	5560	6864	6984	31,082
Maternal death	41	23	21	21	28	134
Maternal mortality ratio (MMR)	669/100,000	414/100,000	377/100,000	305/100,000	400/100,000	431/100,000

**Table II:** Demographic characteristics of maternal deaths.

	2013 (n=41)	2014 (n=23)	2015 (n=21)	2016 (n=21)	2017 (n=28)	Total (n=134)
*Age*						
<20 years	06(14.63%)	03(13.04%)	03(14.28%)	04(19.04%)	04(14.28%)	20(14.92%)
20-24 years	10(24.39%)	05(21.73%)	07(33.33%)	06(28.57%)	07(25%)	35(26.11%)
25-29 years	15(36.58%)	09(39.13%)	07(33.33%)	07(33.33%)	09(32.14%)	47(35.08%)
>30 years	10(24.39%)	06((26.08%)	04((19.04%)	04(19.04%)	08(28.57%)	32(23.88%)
*Parity*						
Primigravida	09(21.95%)	04(17.39%)	06(28.57%)	05(23.80%)	05(17.85%)	29(21.64%)
Multigravida	17(41.46%)	11(47.82%)	09(42.85%)	11(52.38%)	13(46.42%)	61(45.52%)
*Antenatal Care*						
Yes	09(21.95%)	07(30.43%)	06(28.57%)	04(19.04%)	06(21.42%)	32(23.88%)
No	32(78.04%)	16(69.56%)	15(71.42%)	17(80.95%)	22(28.57%)	102(76.11%)

**Table III:** Causes of maternal mortality 2013-2017.

Causes of MMR	2013 (n=41)	2014 (n=23)	2015 (n=21)	2016 (n=21)	2017 (n=28)	Total (n=134)
Hemorrhage	21(51.21%)	12(52.17%)	07(33.33%)	10(47.61%)	14(50%)	64(47.76%)
Hypertensive	11(26.82%)	06(26.08%)	0733.33%)	08(38.09%)	02(7.14%)	34(25.37%)
Thromboembolism	03(7.31%)	02(8.69%)	02(9.52%)	02(9.52%)	01(3.57%)	10(7.46%)
Sepsis	01(2.43%)	01(4.34%)	00	00	04(14.28%	06(4.47%)
Anaesthesia complication	00	00	00	00	01(3.57%)	01(0.74%)
Cardiomyopathy	02(4.87%)	00	2(9.52%)	01(4.76%)	01(3.57%)	06(4.47%)
Status epilepticus	01(2.43%)	00	00	00	00	01(0.74%)
Hepatic failure	02(4.87%)	02(8.69%)	3(14.28%)	00	03(10.71%)	10(7.46%)
Obstructive uropathy	00	00	00	00	01(3.57%)	01(0.74%)
Thyrotoxicosis	00	00	00	00	01(3.57%)	01(0.74%)

## DISCUSSION

In our study, the annual maternal mortality ratio of 431/100,000 was observed over the period of five years. It was quite lower as compared to another part of the country. Studies from Punjab and Sindh have found MMRs in the range of 1017 -2736/100,000 live births.^9^-^12^ South Asian developing countries like India, Pakistan, and Bangladesh have a chief share of maternal deaths throughout the world.^13^ In the yearly trends, maternal deaths fall significantly, from the year 2013 to 2016. However significant cause was found for this positive change.

Women in countryside do not obtain antenatal care and most of the birth take place at home which is attended by inexpert personnel. As observed, non-utilization of antenatal clinic contributes to maternal mortality. Maternal death is on the higher side in antenatal cases 76.11% because of absence of awareness regarding antenatal care, lack of institutional delivery and low availability to healthcare facility. Earlier research has shown that a noteworthy proportion of maternal death and life-threatening complications occurred in those who did not receive antenatal care but referred to the maternity unit in an emergency condition.^14^

Hemorrhage was steadily observed to be the foremost cause of demise in all five years. Over all, it accounted for 47.76% of deaths. It is not a leading contributor in the developing country but also accounts for significant death in developed countries.^15^,^16^ Khan et al.^17^ observe that hemorrhages, infection, organ dysfunction and anemia were among the chief causes of maternal mortality in their studies. In the same study, the authors found mortality were high in cases where delivery was not planned prenatally. In studies from India by Priya et al hemorrhage was the leading cause of maternal mortality of 35.05%.^18^ Though the number of maternal deaths due to hemorrhages has declined because of increased hospital delivery and antenatal care, still it accounts for the maximum maternal deaths. Maternal deaths due to anemia have also decreased due to proper health care facility and nutrition.

Second leading cause of death was hypertension which accounts for 25.37% deaths. In Latin America, hypertensive disorders during pregnancy (25.7%) were the commonest cause of death. In advanced countries, most deaths are due to mainly complication of anesthesia and surgery.^19^ Hypertensive illnesses of pregnancy were the major cause of maternal mortality in studies by Singh et al and Paul et al being 24.01% and 32.6% respectively.^20^,^21^

Pregnancy rises hypercoagulability, stasis, and idleness increase coagulation susceptibility. Among the causes of death of the cases, thromboembolism was the 3rd most frequent cause detected in all cases. thromboembolism is one of the life-threatening causes in pregnant women. Approximately 15% of maternal deaths in developed countries are due to thromboembolism.

Almost 35% of deaths were in the age group between 25-30 years. Our country teenage marriages are common and high fertility rates, high levels of poverty and illiteracy as well as gender discrimination have compounded the situation. In fact, a poor woman is many times more likely to die during childbirth due to malnutrition and anemia.^22^ The lifetime risk of a woman dying due to pregnancy-related causes in developing countries is 1:40 as compared to 1:3600 in the developed world.^23^

## CONCLUSION

There is a decreasing trend of maternal death from 2013 to 2016 but a slight increase was noted in 2017. The maternal mortality ratios remain high. Hemorrhage obstinately contributed as the major causative factor. The findings of the study highlight the need for comprehensive efforts using multisectoral collaborations from stakeholders in reducing maternal mortality.

### Authors’ Contribution

**SR:** Conceived the idea, did data collection & manuscript writing.

**WS:** Did statistical analysis & editing of manuscript.

**SFG:** Did review & final approval of manuscript.
